# Absorption and Bio-Transformation of Selenium Nanoparticles by Wheat Seedlings (*Triticum aestivum* L.)

**DOI:** 10.3389/fpls.2018.00597

**Published:** 2018-05-14

**Authors:** Ting Hu, Huafen Li, Jixiang Li, Guishen Zhao, Wenliang Wu, Liping Liu, Qi Wang, Yanbin Guo

**Affiliations:** ^1^College of Resources and Environmental Sciences, China Agricultural University, Beijing, China; ^2^Beijing Key Laboratory of Biodiversity and Organic Farming, China Agricultural University, Beijing, China; ^3^Beijing Key Laboratory of Diagnostic and Trace Ability Technologies for Food Poisoning, Beijing Center for Disease Prevention and Control, Beijing, China

**Keywords:** HPLC-ICP-MS, selenium nanoparticles, selenium speciation, transformation, uptake, wheat (*Triticum aestivum* L.)

## Abstract

Elemental selenium is one of the dominant selenium species in soil, but the mechanism of its uptake by plants is still unclear. In this study, nanoparticles of elemental selenium (SeNPs) with different sizes were prepared, and their uptake and transformation in wheat (*Triticum aestivum* L.) were analyzed in hydroponic experiments by HPLC-ICP-MS. We found that the SeNPs can be absorbed by wheat seedlings, and the process is energy independent. The addition of aquaporins inhibitor caused 92.5 and 93.4% inhibition of chemosynthesized SeNPs (CheSeNPs) and biosynthesized SeNPs (BioSeNPs) absorption by wheat roots, respectively. The 40 nm SeNPs uptake by wheat roots was 1.8-fold and 2.2-fold higher than that of 140 and 240 nm, respectively. The rate of SeNPs uptake in wheat was much slower than that of selenite [Se (IV)], and CheSeNPs were more efficiently absorbed than BioSeNPs. The SeNPs were rapidly oxidized to Se (IV) and converted to organic forms [selenocystine (SeCys_2_), se-methyl-selenocysteine (MeSeCys), and selenomethionine (SeMet)] after they were absorbed by wheat roots. Additionally, we demonstrated that the aquaporin function in some way is related to the absorption of SeNPs. The particle size and synthesis method of the SeNPs affected their uptake rates by plants. Taken together, our results provide a deep understanding of the SeNPs uptake mechanism in plants.

## Introduction

Selenium (Se) is an essential trace element for mammals, and has important physiological functions such as antioxidation, anticarcinogenic effects, and immunity stimulation (Fairweather-Tait et al., 2011). Selenium is found ubiquitously but is geographically variable in the environment, and is transferred through the geochemical cycle rock-soil-water-plants-mammals (Peters et al., 2016). The daily intake of Se varies geographically, in the world, 0.5–1 billion people have a Se intake below the recommended 55 μg day^−1^ and have been estimated to be Se-deficient, including the people living in south island of New Zealand, and parts of China and Europe (Combs Jr, 2001; Rayman, 2004; Pappas et al., 2008; Lv et al., 2014). Wheat (*Triticum aestivum* L.) is one of the principal cereal grains produced and consumed globally. Supplement of Se into the food chain is one of the effective measures to solve Se deficient situation. Thus, there is potential for it to make a substantially larger contribution of grain Se could be raised to levels found in overseas. Besides that, wheat provides the main source of Se in the world, and people in most European countries such as England and Finland uptake Se from bread (Broadley et al., 2011; Lee et al., 2011).

Selenium is a metalloid element that occurs in different forms, including as selenide [Se (-II)], elemental selenium [Se (0)], selenite [Se (IV)], and selenate [Se (VI)], all of these organic or inorganic Se compounds occur naturally in the environment and accumulates in many organisms (Dissanayake and Chandrajith, 2009). Elemental selenium is one of the dominant Se species in both aerobic and anaerobic soils which contribute 26–66% of the total Se in soil (Fox et al., 2000). Under oxidizing conditions (>300 mV) and moderately reducing conditions (0–200 mV) selenate and selenite will be the most dominant species in soil, respectively (Zhang and Moore, 1996; Martens and Suarez, 1997; Gao et al., 2000). In plant metabolism, selenate is absorbed through a sulfate transporter in the root plasma membrane into the cell, and is then converted into Se-amino acids (Terry et al., 2000; Sors et al., 2005; Schiavon and Pilon-Smits, 2017). Selenite likely enters via a phosphate transport pathway into the plant root, which is a metabolically-dependent active process (Li et al., 2008; Zhang et al., 2013; Kaur et al., 2014). In the process of Se assimilation, selenite is reduced to selenide, and then incorporated into selenocysteine (SeCys). The synthesis of SeCys probably occurs in chloroplasts, the cytosol, and mitochondria, and SeCys can be converted into selenomethionine (SeMet) (White and Broadley, 2009). Plants probably take up organic forms of Se via amino acid permease, and the rate of uptake of SeCys and SeMet by wheat and canola is 20-fold higher than that of selenate or selenite (Schiavon and Pilon-Smits, 2017). However, plants uptake and transformation of elemental Se have not been well study.

Gray and black elemental Se are insoluble and have no bioactivity, while the red selenium nanoparticles (SeNPs) have scavenging effects on different free radicals *in vitro* (Zhang et al., 2001; Huang et al., 2003). SeNPs have found applications in medicine as antimicrobial, antioxidant, and anticancer agents (Hariharan et al., 2012; Forootanfar et al., 2014; Torres et al., 2012; Yang et al., 2012). SeNPs were more effective in increasing the activities of glutathione peroxidase (GSH-Px), thioredoxin reductase (TrxR), and glutathione S-transferase (GST) than SeMet in experiments with rodents and crucian carp, and SeNPs had low cytotoxicity compared with selenite in mice (Shi et al., 2010; Domokos-Szabolcsy et al., 2012; Wadhwani et al., 2016). SeNPs can be chemosynthesized by chemical reducing agents or be biosynthesized by organisms. Compared with chemosynthesized SeNPs (CheSeNPs), the biosynthesized SeNPs (BioSeNPs) are more stable due to natural coating of organic molecules or proteins (Lenz et al., 2011). Currently, the supplementation of plants with Se is usually limited to selenite or selenate, and it is still unclear how plants absorb SeNPs. Previous research has demonstrated that tobacco can take up SeNPs in callus culture and through the root, and that the biological effects of SeNPs are different from selenate in plant tissue culture (Domokos-Szabolcsy et al., 2012).

Here we compared the uptake, translocation and transformation of CheSeNPs and BioSeNPs in wheat seedlings in a controlled hydroponic system. The aim of this work was to identify the key absorption mechanism of SeNPs in wheat seedlings, which has so far remained unclear, and provide a theoretical basis for enhancing the quality and safety of the human diet and ensure the long-term use of new Se resources.

## Materials and methods

### Wheat seedling culture

Wheat (*Triticum aestivum* L. cv. Luyuan 502, Shandong Academy of Agricultural Sciences) caryopses were surface-sterilized with 70% (v:v) ethanol for 90 s, washed three times with deionized water, and germinated in 0.5 mM CaCl_2_ solution at 25°C in the dark. The wheat seedlings were cultured in plastic containers with 2-liter modified 1/5 strength of Hoagland's nutrition solution (1.0 mM KNO_3_, 1.0 mM Ca(NO_3_)_2_, 0.457 mM MgSO_4_, 0.1 mM KH_2_PO_4_, 1.0 μM MnCl_2_, 3 μM H_3_BO_3_, 1 μM (NH_4_)_6_Mo_7_O_24_, 1 μM ZnSO_4_, 0.2 μM CuSO_4_, and 60 μM Fe(III)-EDTA) with 2 mM 2-morpholonoethanesulphonic acid (MES), and the pH was adjusted with KOH to 6.0 (Hogland and Arnon, 1950). The culture solution was renewed every 5 days. The wheat seedlings were grown in a greenhouse under the following conditions: 16 h photoperiod per day with a light intensity at 280 μmol m^−2^s^−2^, 25/18°C day/ night temperature, relative humidity of 60–80%, and 24 h of continuous ventilation.

### Preparation and characterization of SeNPs

Chemosynthesized SeNPs (CheSeNPs) with nominal diameters of 40, 140, and 240 nm were prepared according to previously described methods (Lin and Wang, 2005). Briefly, CheSeNPs were synthesized from selenous acid with sodium thiosulfate (Na_2_S_2_O_3_) as the reducing agent, and sodium dodecyl sulfate (SDS) as a surfactant stabilizer. The CheSeNPs with different particle sizes were collected by centrifugation, washed three times with 10 mM SDS, and suspended with deionized water to 1 mM, respectively. Biosynthesized SeNPs (BioSeNPs) were isolated from *Rahnella aquatilis* HX2 via a modified method described by Dobias et al. (2011). Briefly, HX2 was incubated in Luria-Bertani broth (containing 1% tryptone, 1% NaCl, and 0.5% yeast extract) supplemented with filter-sterilized Na_2_SeO_3_ to a final concentration of 5 mM for 48 h at 28°C. The HX2 cells with SeNPs were collected by centrifugation at 8,000 *g* for 10 min. The precipitates were washed three times with deionized water, suspended with 1 M NaOH solution, boiled for 20 min in a water bath, and amended with a 1/2 volume of n-hexane. The aqueous phase which contained the SeNPs was taken and adjusted the pH to 7.2 using 6 M HCl. The BioSeNPs were collected by centrifugation at 8,000 *g* for 30 min, washed three times and suspended to 1 mM with deionized water for further use.

A transmission electron microscope (TEM) (Hitachi H7500, Tokyo, Japan) and an energy dispersive X-ray (EDX) detector (Hitachi HT7700, Tokyo, Japan) were used to analyze the elemental composition of the CheSeNPs and BioSeNPs. The TEM and EDX analyses were both performed at the National Center for Nanoscience and Technology (Beijing, China) according to the methods of manufacturer's instruction. The SeNPs were observed with the TEM system, and then selected areas within the TEM sections were subjected to elemental composition analysis using an EDX microanalysis system coupled to the TEM. The particle sizes of CheSeNPs and BioSeNPs were measured from manual counting of 200 individual particles from 9 different TEM images, respectively. The voltage was 100 kV and the signal acquisition time was 120 s. The hydrodynamic diameter and zeta potential of the CheSeNPs and BioSeNPs were measured by a dynamic light scattering (DLS) and particle size analyzer (SZ-100, Horiba, Japan).

### Stability and desorption of SeNPs in the wheat root

The roots of 6-week-old wheat seedlings were washed twice with desorption solution (1 mM CaSO_4_, 2 mM MES, pH 6.0) for 30 min, and transferred to 100 ml 2 mM MES (pH 6.0) solution with 5 μM CheSeNPs. Each treatment was replicated three times. After 60 min absorption, the root about 1 g treated with SeNPs was washed three times by 10 ml desorption solution. The Se concentrations in washed desorption solution were detected.

Pretreated wheat seedlings were transferred to 300 ml containers (two plants per container) with 100 ml 5 μM CheSeNPs or BioSeNPs. Solutions containing 5 μM CheSeNPs or BioSeNPs without plants served as controls. Each treatment was replicated three times. Two milliliter aliquots were collected at 0, 2, 4, 8, 16, and 24 h during the incubation, filtered through 0.22-μm mixed cellulose nitrate filters (Millipore, Billerica, MA, USA), and stored at 4°C for selenite analysis.

### Kinetics of SeNPs absorption

The roots of 6-week-old wheat seedlings were washed twice with desorption solution (1 mM CaSO_4_, 2 mM MES, pH 6.0) for 30 min, and the plants were transferred to 100 ml 2 mM MES (pH 6.0) solution with a series Se concentrations (0, 0.1, 0.5, 1.0, 5.0, 10.0, 20.0 μM selenite, CheSeNPs, and BioSeNPs, respectively). Each treatment was replicated three times (two plants per pot), and the experiment was repeated three times. After 60 min absorption, the roots were rinsed with deionized water and then transferred into ice-cold desorption solution for 15 min with three times to remove the Se adhering to the root surfaces. Then, the shoots and roots of wheat seedlings were separated, lyophilized and analyzed for Se.

### Effect of a respiratory inhibitor on SeNPs uptake

The respiratory inhibition assay was performed according to the reported method (Li et al., 2008) with slight modification. Carbonyl cyanide between chlorobenzene hydrazone (CCCP) known as a protonophore or uncoupler of oxidative phosphrylation was used as a respiratory inhibitor in this experiment (Volkov et al., 1988). Wheat seedlings were pretreated as described above, and then the roots were transferred to 100 ml 2 mM MES (pH 6.0) solution with 5 μM CheSeNPs or BioSeNPs, with or without 1 μM carbonyl cyanide between chlorobenzene hydrazone (CCCP), which was initially dissolved in ethanol; the final ethanol concentration was 0.01% (v/v). Solutions containing 5 μM CheSeNPs or BioSeNPs with 0.01% (v/v) ethanol served as controls. Each treatment was replicated three times (two plants per pot), and the experiment was repeated three times. After 60 min absorption, the wheat roots were treated as described above in the kinetics section.

### Effect of aquaporin inhibition on SeNPs uptake

The aquaporin inhibition assay was performed according to the reported method (Zhang et al., 2006) with slight modification. Wheat seedlings were prepared as described above in the kinetics section, then transferred to 100 ml 2 mM MES (pH 6.0) solution with 5 μM CheSeNPs or BioSeNPs, with or without 0.1 mM AgNO_3_. Each treatment was replicated three times (two plants per pot), and the experiment was repeated three times. After 60 min absorption, the wheat seedlings were treated as described above.

### Effect of particle size on SeNPs uptake

Pretreated wheat seedlings were transferred to 100 ml 2 mM MES (pH 6.0) solution with 5 μM 40, 140, or 240 nm CheSeNPs. Each treatment was replicated three times (two plants per pot), and the experiment was repeated three times. After 60 min absorption, the wheat seedlings were treated as described above.

### Uptake and transfer of SeNPs in wheat

Prepared wheat seedlings were transferred to 100 ml 2 mM MES (pH 6.0) solution with 5 μM selenite, CheSeNPs, or BioSeNPs. Each treatment was replicated three times (two plants per pot). The seedlings were harvested at 24, 48, and 72 h and treated as described above. To evaluate the transfer potential of Se from roots to shoots, the transfer factor (TF) (defined as the ratio of Se concentration in shoots to roots) was calculated (Huang et al., 2015).

### Total selenium analysis

The total Se concentration in the treated wheat tissues was determined with a AFS-920 hydride generation flame atomic fluorescence spectrometer (HG-AFS) (Beijing Jitian Analysis Instruction Co., Beijing, China). Powdered seedling samples (250 mg) were digested with 8 ml 15.3 M HNO_3_ under the microwave digestion program described by Baldwin et al. (1994). After cooling down, 2.5 ml 6 M HCl was added to each tube, and the contents were heated to 100°C for 1 h to reduce selenate to selenite (Zhang et al., 2006). The obtained solutions were diluted with deionized water to a final volume of 50 ml. Two milliliter samples were injected into the HG-AFS system, and each sample was prepared and analyzed in triplicate for linear estimation based on regression analysis of the effects of Se treatments using Microsoft Excel. Blanks and a certified reference material (Chinese cabbage material, GBW 10014) were included in each batch of samples for quality control. The recovery for GNW-10014 was 85.2–119.7%.

### Selenium species transformation and analysis

Prepared wheat seedlings were transferred to 100 ml 2 mM MES (pH 6.0) solution with 10 μM selenite, CheSeNPs, or BioSeNPs. Each treatment was replicated three times (two plants per treatment). After 24 h absorption, the shoots and roots were separated, lyophilized and analyzed for Se species.

A powdered sample (250 mg) was mixed with 8 mg ml^−1^ protease XIV (Sigma Chemical Co., St. Louis, MO, USA), and shaken for 24 h at 37°C, filtered through a filter paper and then through 0.22-μm mixed cellulose nitrate filters. Samples of nutrient solution were diluted appropriately and filtered through 0.22-μm mixed cellulose nitrate filters.

The Se species were separated using an anion-exchange column (Hamilton PRP-X100) in conjunction with ICP-MS (Table S1). The 100 μl samples were injected into an HPLC-ICP-MS system (Agilent LC 1260 series and ICP-MS 7700; Agilent Technologies, Santa Clara, CA, USA) and eluted with 30 mM (NH_4_)_2_HPO_4_ (pH 6.0) at a flow rate of 1 ml min^−1^. Peaks were identified according to the retention times of standard compounds [i.e., selenocystine (SeCys_2_), Se-methyl-selenocysteine (MeSeCys), Se (IV), SeMet, and Se (VI)] purchased from the National Research Center for Certified Reference Materials, Beijing, China. The identified Se species were quantified based on the peak areas of the calibration curves using an HPLC workstation.

### Statistical analysis

All analyses were performed in triplicate, and the results were expressed as mean values and standard error (SE). The data were processed using SigmaPlot 12.0. One-way ANOVA with multiple comparisons using Duncan's test was employed to compare the means among different treatments (*p* < 0.05) using the SAS software (version 9.0; SAS Inc., Cary, NC, USA).

## Results

### SeNPs characterization

To verify the SeNPs produced by chemosynthesis and biosynthesis, the characteristics of the SeNPs were examined using a TEM and an EDX detector. Under the TEM, the prepared NPs synthesized by chemical and biological methods both appeared as well-dispersed spherical particles with average diameters of 140 ± 10 nm and 140 ± 40 nm (Figures S1A,C), respectively. The hydrodynamic diameter of the CheSeNPs and BioSeNPs were carried out by DLS, as shown in the Figures S2A,B. The hydrodynamic diameter of the CheSeNPs and BioSeNPs was 157.8 ± 30.6 nm and 151.6 ± 41.9 nm, respectively (Figure S2). The zeta potential of CheSeNPs and BioSeNPs were −46.5 and −51.1 mV, respectively (Figure S3). The EDX results demonstrated that the NPs had specific absorption peaks for Se at 1.37 (peak SeLα), 11.22 (peak SeKα), and 12.49 (peak SeKβ) keV (Figures S1B,D). These results indicated that the chemosynthesized and biosynthesized NPs were SeNPs (Figure S1).

### SeNPs absorption by wheat roots

To verified the elutability of the desorption solution, the desorption experiment was preformed. The Se concentration in the first two times washed desorption solution was 30.8 ± 0.1 μg L^−1^ and 4.2 ± 0.1 μg L^−1^, respectively (Figure S4). In the third washed desorption solution, selenium could not be detected (Figure S4). This result indicated that the desorption solution could remove SeNPs adhered on roots surface efficiently.

**Figure 1:**
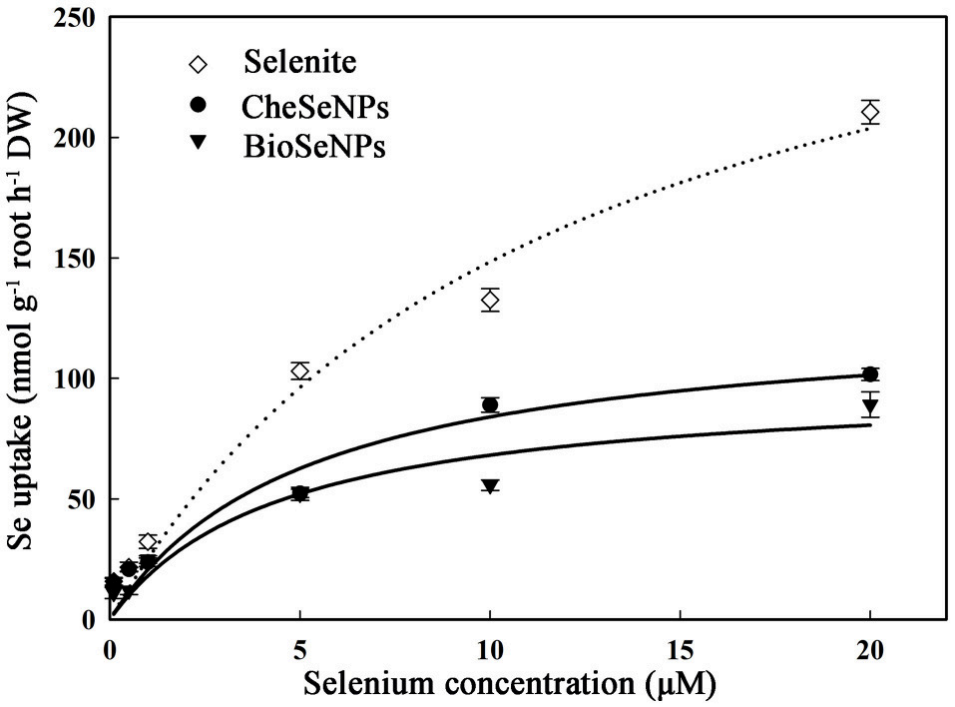
Concentration-dependent kinetics for selenite, CheSeNPs, and BioSeNPs absorption within 60 min. Data are means ± standard error (SE) (*n* = 3). The curves represent the fitted Michaelis-Menten kinetics.

To assess the absorption of selenite, CheSeNPs, and BioSeNPs by plants, the absorption kinetics were assayed using a series of Se concentrations in hydroponic experiments. The Michaelis-Menten equations for selenite, CheSeNPs, and BioSeNPs influx into wheat roots are shown in Figure 1 and Table 1. The *V*_max_ for selenite influx was 2.5- and 3.3-fold higher than for CheSeNPs and BioSeNPs, and the K_m_ value of selenite was 2.3- and 2.6-fold higher than for CheSeNPs and BioSeNPs, respectively (Table 1). SeNPs absorption by wheat roots increased with the external SeNPs concentration and reached a plateau at 10 μM of Se, while selenite absorption into wheat roots increased with the external selenite concentration, but did not reach a plateau in this experiment (Figure 1).

**Table 1:** Concentration-dependent kinetics parameters for selenite, CheSeNPs, and BioSeNPs influx into wheat (*Triticum aestivum* L.) roots within 60 min.

**Uptake kinetics parameters**	**Different treatments**		
	**Selenite**	**CheSeNPs**	**BioSeNPs**
*V*_max_ (nmol g^−1^ root h^−1^)	324.7 ± 58.0	127.6 ± 21.4	98.5 ± 17.9
*K*_m_ (μM)	11.9 ± 4.9	5.2 ± 2.5	4.5 ± 2.5
*R*^2^	0.9787	0.9425	0.9236

*Kinetic parameters were calculated from mean Se influx (n = 3) using Michaelis-Menten function nonlinear regression model*.

### SeNPs uptake was unaffected by CCCP and disrupted by AgNO_3_

**Figure 2:**
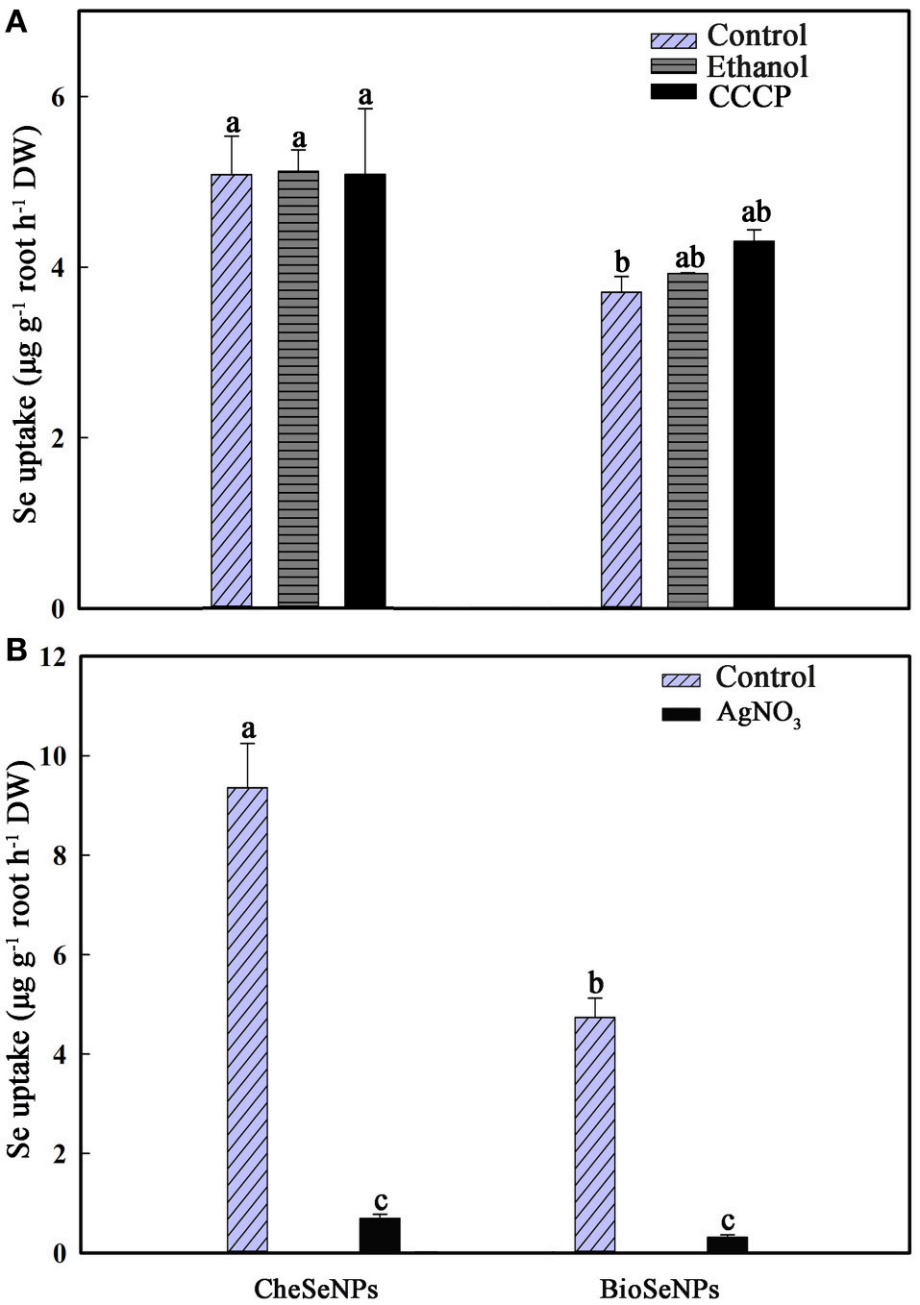
**(A)** Effect of the metabolic inhibitor carbonyl cyanide m-chlorophenylhydrazone (CCCP) on the influx of CheSeNPs and BioSeNPs into wheat (*Triticum aestivum* L.) roots within 60 min. **(B)** Effect of the aquaporin inhibitor AgNO_3_ on the influx of CheSeNPs and BioSeNPs into wheat roots. SeNPs were supplied at 5 μM. Data are means ± SE (*n* = 3); different letters differ significant at *p* = 0.05 (Duncan's test).

CCCP is known as a protonophore or uncoupler of oxidative phosphrylation, which is believed to be inhibition of ATP formation by uncoupling oxidative phosphorylation (Volkov et al., 1988). Thus, it can be used to eliminate the effect of root respiration. To determine whether the uptake of SeNPs by wheat was a passive diffusion process or consumed energy, 1 μM CCCP was included in the experiment. The uptake of CheSeNPs and BioSeNPs was measured after wheat roots were exposed to the two Se species for 60 min. Compared with the control treatment, the addition of ethanol or CCCP to the nutrient solution did not significantly affect the uptake of CheSeNPs or BioSeNPs (Figure 2A). To examine the uptake of SeNPs by plants through water channels, 0.1 mM AgNO_3_ was used as a water channel blocker (Niemietz and Tyerman, 2002). The AgNO_3_ treatment resulted in 92.5 and 93.4% inhibition of CheSeNPs and BioSeNPs uptake, respectively (Figure 2B); these results indicated that the uptake of SeNPs could be disrupted by AgNO_3_.

### SeNPs uptake was correlated with particle sizes

**Figure 3:**
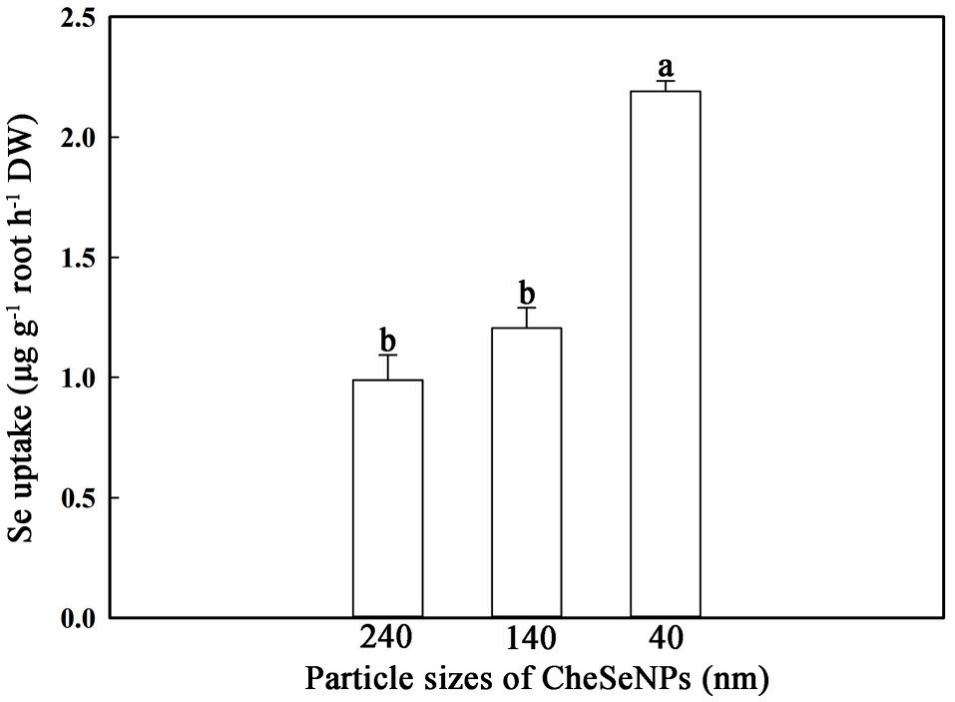
Accumulation of CheSeNPs of different sizes in wheat (*Triticum aestivum* L.) roots within 60 min. Data are means ± SE (*n* = 3); different letters indicate significant difference at *p* = 0.05 (Duncan's test).

SeNPs of different sizes were prepared, and the effects of particle size on SeNPs uptake by wheat were analyzed. The results indicated that the SeNPs uptake by wheat roots was affected by the particle size in the CheSeNPs treatments (Figure 3). The Se absorption by wheat roots under 40 nm CheSeNPs treatment was significantly increased compared with 140 and 240 nm CheSeNPs treatments, being 1.8- and 2.2-fold higher, respectively. There was non-significant difference between the 140 and 240 nm CheSeNPs treatments.

### Selenium accumulation and translocation in wheat

To confirm that the wheat absorbed the SeNPs and not other Se species in the experimental system, the stability of SeNPs in the plant culture solution with or without wheat seedlings was monitored. The SeNPs concentration remained steady during the wheat absorption process (Figure S5). The measured concentrations of selenite accounted for 2.2 and 0.5% of the CheSeNPs- or BioSeNPs-treatment concentrations in the initial solution, respectively (Figure S5). The concentration of selenite in the CheSeNPs or BioSeNPs plus wheat root treatments did not significantly change with treatment time (Figure S5). Additionally, the selenite concentration did not markedly increase with exposure time in the solutions of CheSeNPs or BioSeNPs alone (without wheat seedlings) (Figure S5).

**Figure 4:**
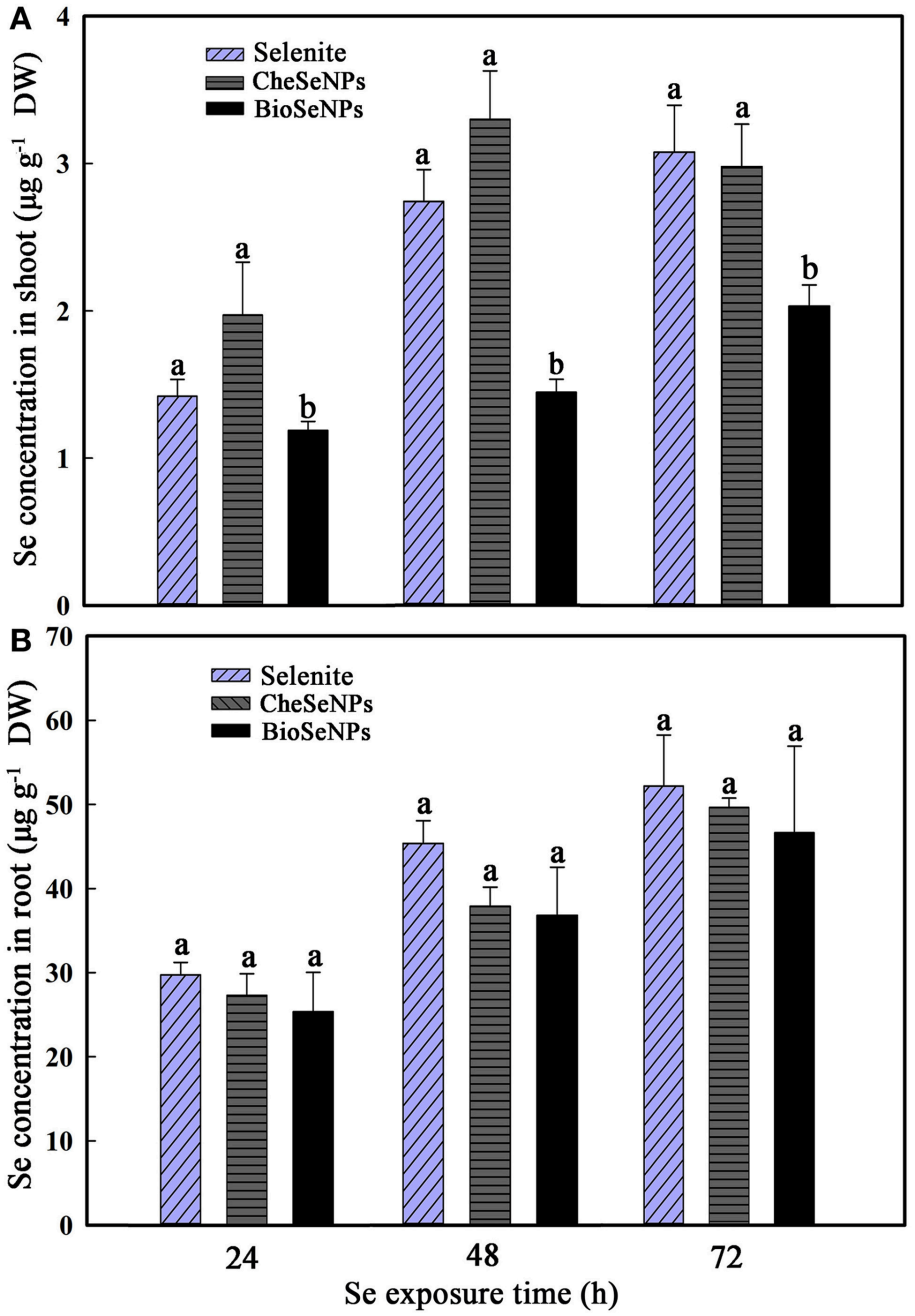
Selenium accumulation in wheat (*Triticum aestivum* L.) **(A)** shoots and **(B)** roots in response to selenite, CheSeNPs, and BioSeNPs treatments for 24, 48, and 72 h. Data are means ± SE (*n* = 3); different letters indicate significant difference at *p* = 0.05 (Duncan's test).

The uptake and translocation of Se in wheat under selenite, CheSeNPs, and BioSeNPs treatments was detected by HG-AFS. The Se concentration in wheat roots did not show a significant difference among the selenite, CheSeNPs, and BioSeNPs treatments at 24, 48, and 72 h (Figure 4B). However, the concentration of Se in wheat roots significantly increased (*p* < 0.05) with the exposure duration from 24 to 72 h under all treatments. Generally, the Se concentration in wheat roots and shoots increased with increasing exposure time (Figure 4). In the BioSeNPs treatment, the Se concentration in wheat shoots was significantly lower than in the CheSeNPs and selenite treatments, whereas there was no significant difference in the Se concentration between the CheSeNPs and selenite treatments (Figure 4A). Compared with the selenite treatment, the Se concentration decreased by 16.3–47.3% in BioSeNPs-treated wheat shoots, but showed a slight increase in the CheSeNPs treatment at earlier exposure times (24 and 48 h).

**Figure 5:**
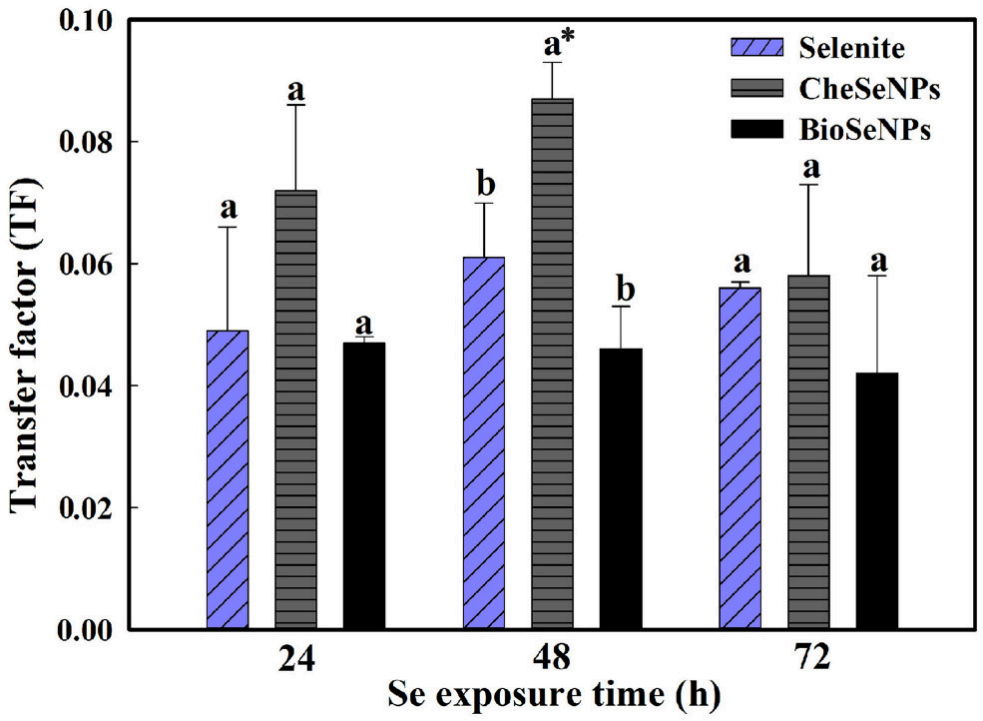
Selenium transfer factor (TF) in wheat under selenite, CheSeNPs, and BioSeNPs treatments for 24, 48, and 72 h. Data are means ± SE (*n* = 3); different letters indicate significant at *p* = 0.05 (Duncan's test); * differs significantly at *p* = 0.011.

The TF, defined as the ratio of the Se concentration in shoots to that in roots, can be used to evaluate the transfer potential of Se from roots to shoots. The TF values in all treatments were generally below 0.1, which indicated that, like selenite, BioSeNPs, and CheSeNPs had low translocation rates from roots to shoots (Figure 5). Among the treatments, BioSeNPs showed the lowest translocation rate and CheSeNPs the highest. The TF value of the CheSeNPs treatment at 48 h was 1.4 and 1.9 times higher than those of the selenite and BioSeNPs treatments, respectively (Figure 5). However, there was no significant difference in the TF values between the treatments at 24 and 72 h.

### Selenium species in wheat seedlings

**Figure 6:**
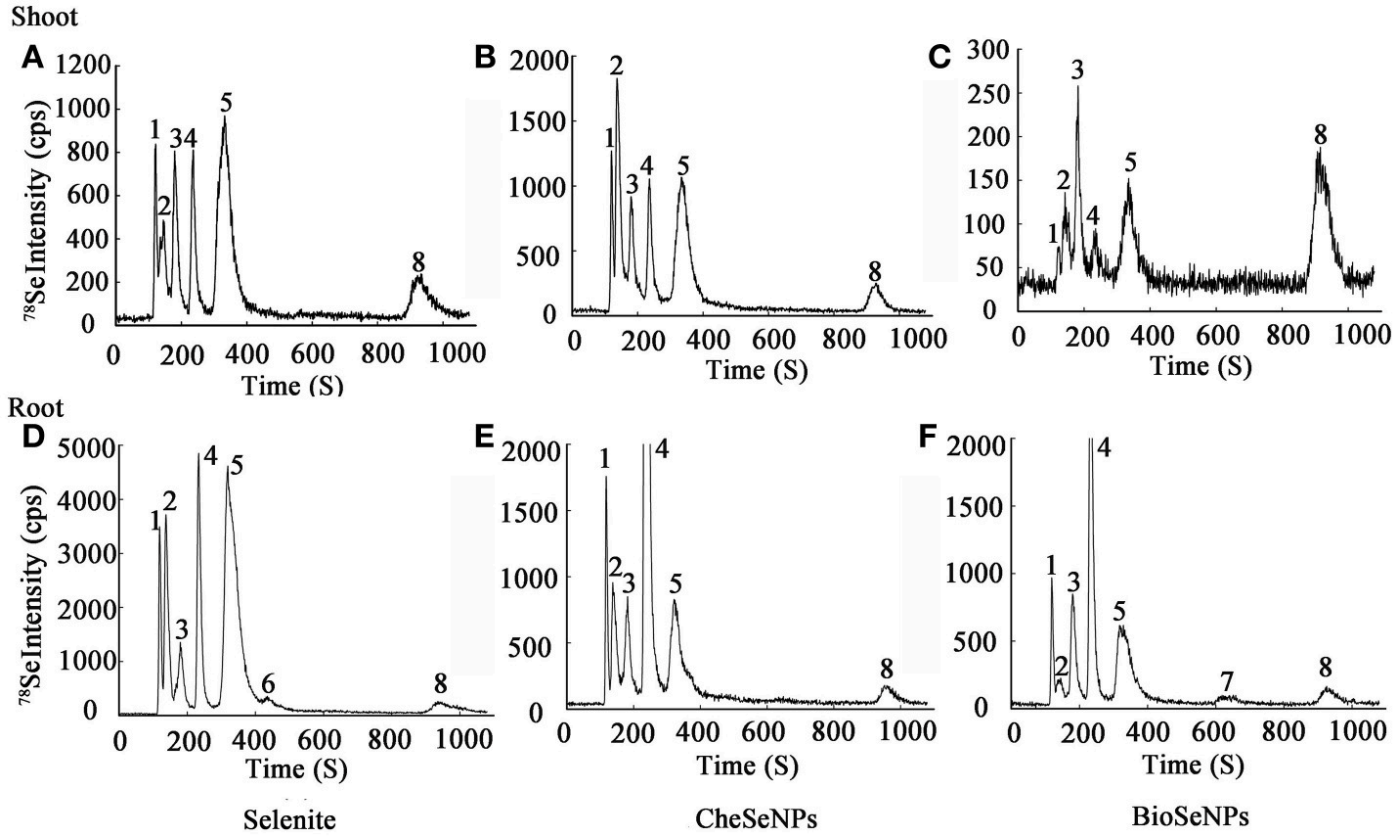
Examples of chromatograms of selenium (Se) speciation in protease XIV extracts of wheat (*Triticum aestivum* L.) roots and shoots, as determined by anion exchange HPLC-ICP-MS. The intensity (count per second, cps) is for m/z 78. **(A–C)** Se species in wheat shoots under selenite, CheSeNPs, and BioSeNPs treatment, respectively. **(D–F)** Se species in wheat roots under selenite, CheSeNPs, and BioSeNPs treatment, respectively. 1 (Unknown Se species); 2 (SeCys_2_, Selenocystine); 3 (MeSeCys, Se-methyl-selenocysteine); 4 [Se (IV), Selenite]; 5 (SeMet, Selenomethionine); 6 (Unknown Se species); 7 (Unknown Se species); 8 [Se (VI), Selenate].

Selenium species were extracted from wheat root and shoot tissues by enzymatic hydrolysis and detected with HPLC-ICP-MS. The analysis results showed that the BioSeNPs and CheSeNPs were absorbed and converted to organic forms by wheat plants (Figure 6). In total, eight Se species were observed, but only five were identified, including SeCys_2_, MeSeCys, SeMet, Se (IV), and Se (VI). SeCys_2_, MeSeCys, Se (IV), Se (VI) and an unidentified peak (1) eluted at a retention time of 116 s (RT _116_) were detected in wheat roots and shoot tissues under all treatments (Figure 6).

HPLC-ICP-MS analysis revealed that the extraction procedure extracted 50.2–60.1% of the total Se from the roots and shoots of the treated wheat, except in the shoot under BioSeNPs treatment (33.4%) (Table S2). In the wheat roots, Se (IV) was the dominant species under the CheSeNPs- (34.0% of the total Se) and BioSeNPs treatments (17.6%) whereas SeMet was the dominant Se species (18.0%) under the selenite treatment and was found at a concentration of 8.71 μg g^−1^. Se (VI) was also present in wheat roots under all treatments, but had a relatively lower concentration, representing only <2.5% of the total Se (Figure 6, Table 2). In the root extracts, an unidentified peak (7) was eluted at a retention time of 637 s (RT _637_) for the BioSeNPs treatment plants, whilst another unidentified peak (6) was eluted at a retention time of 435 s (RT _435_) for the selenite treatment plants (Figure 6). The Se speciation in wheat shoots showed difference to that in roots. SeCys_2_ (11.4%) was the dominant species in wheat shoots for CheSeNPs treatment plants, whilst Se (VI) accounted for 7.4% of the Se in the BioSeNPs treatment plants. In the selenite treatment plants, the most abundant organic Se compound was SeMet, which occupied 18.4% of the total Se in wheat shoots (Figure 6, Table 2).

**Table 2:** Effects of selenium (Se) species supplied on Se speciation in protease XIV extracts from wheat (*Triticum aestivum* L.) roots and shoots.

**Species**		**Se species supplied**					
		**Selenite**		**CheSeNPs**		**BioSeNPs**	
		**μg g^−1^ DW**	Percentage %^*^	**μg g^−1^ DW**	**Percentage %**	**μg g^−1^ DW**	**Percentage %**
Shoot	SeCys_2_	0.33 ± 0.06*b*	3.0 ± 0.6	1.48 ± 0.06*a*	11.4 ± 0.6	0.08 ± 0.01*b*	2.3 ± 0.1
	MeSeCys	0.24 ± 0.08*b*	2.2 ± 0.8	0.28 ± 0.05*c*	2.2 ± 0.4	0.07 ± 0.04*b*	2.0 ± 0.5
	Se (IV)	0.22 ± 0.15*b*	2.0 ± 1.6	0.38 ± 0.01*c*	2.9 ± 0.1	0.06 ± 0.02*b*	1.7 ± 0.3
	SeMet	2.05 ± 0.73*a*	18.4 ± 0.7	1.01 ± 0.11*b*	7.8 ± 1.0	0.19 ± 0.04*a*	5.4 ± 0.6
	Se (VI)	0.25 ± 0.04*b*	2.2 ± 0.5	0.22 ± 0.00*c*	1.7 ± 0.1	0.26 ± 0.01*a*	7.4 ± 0.1
Root	SeCys_2_	2.36 ± 1.09*ab*	8.0 ± 2.7	2.12 ± 0.13*b*	6.6 ± 2.1	0.11 ± 0.03*b*	0.8 ± 0.1
	MeSeCys	0.90 ± 0.21*ab*	3.0 ± 0.5	0.85 ± 0.04*c*	2.6 ± 0.9	0.32 ± 0.07*b*	2.4 ± 0.3
	Se (IV)	3.55 ± 1.02*ab*	11.7 ± 2.5	11.00 ± 0.15*a*	34.0 ± 6.8	2.36 ± 1.00*a*	17.6 ± 3.6
	SeMet	5.45 ± 2.27*a*	18.0 ± 5.5	1.87 ± 0.13*b*	5.8 ± 1.7	0.67 ± 0.22*b*	5.0 ± 0.8
	Se (VI)	0.40 ± 0.10*b*	1.3 ± 0.2	0.33 ± 0.06*d*	1.0 ± 0.4	0.34 ± 0.14*b*	2.5 ± 0.5

*Mean values (n = 3) ± standard error (SE). Selenite, sodium selenite; CheSeNPs, chemogenic selenium nanoparticels; BioSeNPs, biogenic elemental selenium nanoparticles; SeCys_2_, selenocystine; MeSeCys, Se-methyl-selenocysteine; Se (IV), selenite; SeMet, selenomethionine; Se (VI), selenate*.

* *The percentage was calculated by the concentration of Se species / the concentration of Se in tissues × 100%. The different letters indicate statistically significant differences between the treatments as the p < 0.05*.

## Discussion

### Mechanisms of SeNPs uptake

On account of most NPs have a proportionately very large surface area and this surface can have a high affinity for plants roots (Cheng et al., 2004; Aslani et al., 2014). The Figure S4 indicated that the desorption solution could remove SeNPs adhered on roots surface efficiently. In addition, the results in Figure 2 will also give evidence to SeNPs desorption from roots. If the SeNPs could not be removed from root efficiently by desorption solution, Se concentration of root in AgNO_3_ treatment could not significantly decreased compared to the control.

The phytouptake of several metal nanoparticles including Se has been investigated, and these studies have demonstrated that nanoparticles can adhere to plant roots and induce chemical or physical uptake (Domokos-Szabolcsy et al., 2012; Aslani et al., 2014). However, the uptake mechanism of SeNPs is not well understood. The uptake kinetics of selenite, CheSeNPs, and BioSeNPs showed that CheSeNPs and BioSeNPs influx into wheat roots was much slower than selenite influx (Figure 1, Table 1). The lower *V*_max_ and *K*_m_ values for both CheSeNPs and BioSeNPs suggested a distinct difference in the uptake and affinity for these Se species compared with selenite.

A previous study reported that the metabolic inhibitor CCCP inhibited the uptake of selenite by 80%, and demonstrated that selenite uptake was a metabolically-dependent active process (Li et al., 2008). However, neither the uptake of CheSeNPs nor BioSeNPs was sensitive to the metabolic inhibitor CCCP (Figure 2A). CCCP is used as an oxidative phosphorylation inhibitor to reduce the operation of the ATP synthase, and eliminate the effect of wheat root respiration (Volkov et al., 1988). Thus, it is likely that wheat root absorption of SeNPs is a passive diffusion process, and does not consume energy. Furthermore, our results demonstrated that AgNO_3_ significantly disrupted the wheat root absorption of CheSeNPs, and BioSeNPs (Figure 2B). AgNO_3_ is a potential inhibitor of aquaporins of plant origin (Niemietz and Tyerman, 2002). The mechanism of AgNO_3_ inhibits aquaporin function is that the pore diameter of an aquaporin is 2.8 Å and the ionic radius of Ag^+^ is 2.5 Å. Hence elements with ionic radii similar to silver may be potent inhibitors of aquaporins. Silver reacts with the sulfhydryl group of a cysteine and also with a histidine, resulting in gating by the targeted aquaporin (Niemietz and Tyerman, 2002). The inhibition of CheSeNPs and BioSeNPs uptake by AgNO_3_ was due to its effect on aquaporins. It was reported that AgNO_3_ blocked the water channels and inhibited selenite absorption by rice roots at low pH by 73% (Zhang et al., 2006). Earlier literature has documented that water channels do not completely exclude small uncharged molecules, and they can transmit considerable amounts of water as solutes diffuse across channels (Hertel and Steudle, 1997). The aquaporins inhibiter resulted in 92.5 and 93.4% inhibition of CheSeNPs and BioSeNPs uptake, respectively (Figure 2B). The diameter of aquaporins is about 2.8 Å, it seems too small for SeNPs to pass though (Niemietz and Tyerman, 2002). The aquaporins play important roles in the physiological processes, including transport of water, mineral nutrition, carbon, nitrogen fixation, promote cell elongation, differentiation, and stomata movement (Maurel et al., 2008). Thus, we speculate that the inhibiter of aquaporin function in some way influenced the wheat seedlings absorb of SeNPs.

Evidences have suggested that only NPs or NPs aggregates with a diameter less than the pore diameter of the cell wall can easily pass through and reach the plasma membrane (Moore, 2006; Zhang et al., 2008; Dietz and Herth, 2011). Because of the limited sizes of the cells pore, only NPs smaller than 5 nm may traverse an intact cell wall (Carpita et al., 1979; Li et al., 2015). As the size of SeNPs is greater than the pore size of cell walls, this hypothesis is refuted by the results of the present study. This study demonstrated that smaller CheSeNPs were more easily absorbed than larger ones (Figure 3). Thus, the uptake of SeNPs is not solely dependent on the pore diameter of the cell wall; other mechanisms may be involved. Furthermore, NPs below 100 nm in size possess unique physico-chemical, electrical, optical, and biological activities, compared with their bulkier counterparts (Ingle et al., 2014).

### Transformation of SeNPs in wheat plants

The CheSeNPs and BioSeNPs were synthesized using selenite in our experiments (Figure S1). The CheSeNPs and BioSeNPs have a relatively higher zeta potential (−46.5 and −51.1 mV, respectively) in water solution and thus form a stable dispersion, enabling the application possible for plant. We monitored the dynamics of Se concentrations in the solutions in the SeNPs treatments with or without wheat seedlings, and found little (0.05–2.2%) selenite in the initial treatment solution. The selenite concentration did not significantly increase during the experiment, which indicated that the SeNPs appeared stable and were not oxidized to selenite in the solution (Figure S5). The results indicated that the plants absorbed the SeNPs and not the selenite in the experiment.

The selenium species were extracted with protease XIV. Because of the limited availability of standard Se compounds, the SeCys_2_, MeSeCys, Se (IV), SeMet, Se (VI) were quantitatively and qualitatively measured in this study (Figure 6). Unknown compounds and the low extraction efficiencies accounted for the difference between the total Se content and the sum of the identified peaks. Previous studies have demonstrated that inorganic Se (i.e., selenate and selenite) can be taken up by plants and then transformed into organic Se compounds (i.e., SeCys_2_, SeMet, and MeSeCys) and bound within proteins (Vogrincic et al., 2009; Schiavon and Pilon-Smits, 2017). However, little information is available on SeNPs transformation in plants. Our study showed that SeNPs could also be taken up by plants and then transformed into organic Se compounds, selenite, and selenate in roots and shoots, which demonstrated the the bioavailability of SeNPs in plants. The root extracts contained a number of Se species with Se (IV) being the dominant species in wheat roots under the CheSeNPs and BioSeNPs treatments (Table 2). By contrast, when wheat plants were supplied with selenite, it was rapidly converted to organic forms in the roots, although a tiny portion of selenite was remained. Similarly, Li et al. (2008) found that selenite supplied to wheat roots was rapidly assimilated into organic forms and detected a relatively small concentration of Se (IV) in wheat root extracts. Because Se (IV) was the most abundant species in the root extracts of CheSeNPs- and BioSeNPs-treated plants, we hypothesize that the oxidation reactions occurred in the root cells. In wheat shoots, SeMet (18.4%) was the dominant species in the selenite treatment plants, SeCys_2_ (11.4%), and SeMet (7.8%) were the main species in the CheSeNPs treatment plants, and Se (VI) (7.4%) and SeMet (5.4%) were the main species in the BioSeNPs treatment plants (Table 2). The transformation of Se species in plants is a dynamic process, and SeCys, selenocystathionine, selenohomoserine, and SeMet are all considered likely intermediates in the assimilation of selenite into selenoproteins (Shrift, 1969). Duncan et al. (2016) reported that SeMet was the major Se species in wheat grain samples, and Vogrincic et al. (2009) found that SeMet and MeSeCys were the most abundant species in Se-enriched plants. Another interesting phenomenon was that under the BioSeNPs treatment, the percentage of Se (VI) was 7.4%, which was 4.4- and 3.4-fold higher than that in the CheSeNPs and selenite treatments, respectively (Table 2). This suggests that some complex metabolic process occurs in plants based on the special biological characteristics of BioSeNPs. In contrast to the synthesis of CheSeNPs, BioSeNPs are synthesized by complex microbial communities and capping agents (i.e., proteins, polysaccharides, phenols, amines and alcohols) are present on the surfaces of BioSeNPs (Dwivedi et al., 2011; Husen and Siddiqi, 2014; Jain et al., 2015). The BioSeNPs synthesized by HX2 which coated two main proteins *Rahnella aquatilis* flagellin C (FliC) and the outer membrane protein porin (Data no shown). Thus, the extracellular polymeric substances of BioSeNPs may be one of the unfavorable factors influencing their transport and accumulation in wheat plants.

### SeNPs translocation from roots to shoots

The TF, defined as the ratio of Se concentration in shoots to roots is generally used to evaluate the translocation potential of Se from plant roots to shoots (Huang et al., 2015). CheSeNPs and selenite showed similar translocation of Se from roots to shoots during the longer exposure period (72 h), while the BioSeNPs were rarely translocated to shoots (Figure 4). The TF values were below 0.1 in all treatments, which indicated that little SeNPs and selenite were transported to the shoots. The low TFs of SeNPs and selenite from roots to shoots may be due to their rapid conversion to organic Se compounds (SeCys_2_, MeSeCys, and SeMet) and retention in wheat roots. The shape, size, chemical composition, concentration, surface structure, aggregation and solubility of NPs are critical factors influencing the plant uptake (Aslani et al., 2014).

In conclusion, the present study has provided physiological evidence that SeNPs can be actually absorbed by wheat seedlings. SeNPs influx into wheat roots is a passive diffusion process, and the uptake rate of SeNPs is lower than for selenite. CheSeNPs below 50 nm in size were more easily absorbed into wheat roots. The aquaporin function is in some way involved in the SeNPs uptake by wheat seedlings. Few SeNPs were transported from roots to shoots, and they were rapidly assimilated into Se (IV) and organic forms in both wheat roots and shoots. Thus, water management and smaller diameter of SeNPs produced by microorganisms may improve the wheat absorption of SeNPs in the field. Thus, SeNPs could be used as a new fertilizer to produce Se-biofortified plants, which might improve Se supplementation for humans and domestic animals.

## Author contributions

YG and HL: Conceived and designed the experiments; TH, JL, and QW: Performed the experiments; YG, HL, and TH: Analyzed the data; GZ, WW, and LL: Contributed reagents, materials, and analysis tools; YG, HL, and TH: Wrote the paper.

### Conflict of interest statement

The authors declare that the research was conducted in the absence of any commercial or financial relationships that could be construed as a potential conflict of interest.

## Supplementary material

Figure S1 TEM images of **(A)** CheSeNPs [the ratio of the concentration of sodium selenite (Na_2_SeO_3_) to sodium thiosulfate (Na_2_S_2_O_3_) is 1:4] and **(C)** BioSeNPs (formed by strain HX2 with 15 mM Na_2_SeO_3_ for 48 h) and EDX spectra of the chemosynthesized nanoparticles **(B)** and biosynthesized nanoparticles **(D)**.

Figure S2 Hydrodynamic diameter of **(A)** CheSeNPs and **(B)** BioSeNPs.

Figure S3 Zeta potential of **(A)** CheSeNPs and **(B)** BioSeNPs.

Figure S4 Concentration of Se in the desorption solution with the wash times.

Figure S5 Selenite concentration in SeNPs treatments solutions (CheSeNPs; BioSeNPs).

Table S1 ICP-MS parameters for analysis of Se species in wheat roots and shoots.

Table S2 Extraction efficiency of selenium in wheat roots and shoots by protease XIV.
