# Supplementary figures and images for: Absorption and Bio-Transformation of Selenium Nanoparticles by Wheat Seedlings (Triticum aestivum L.)
Source: Front Plant Sci. 2018 May 14;9:597. doi: 10.3389/fpls.2018.00597 (PMC5960721; doi:10.3389/fpls.2018.00597)

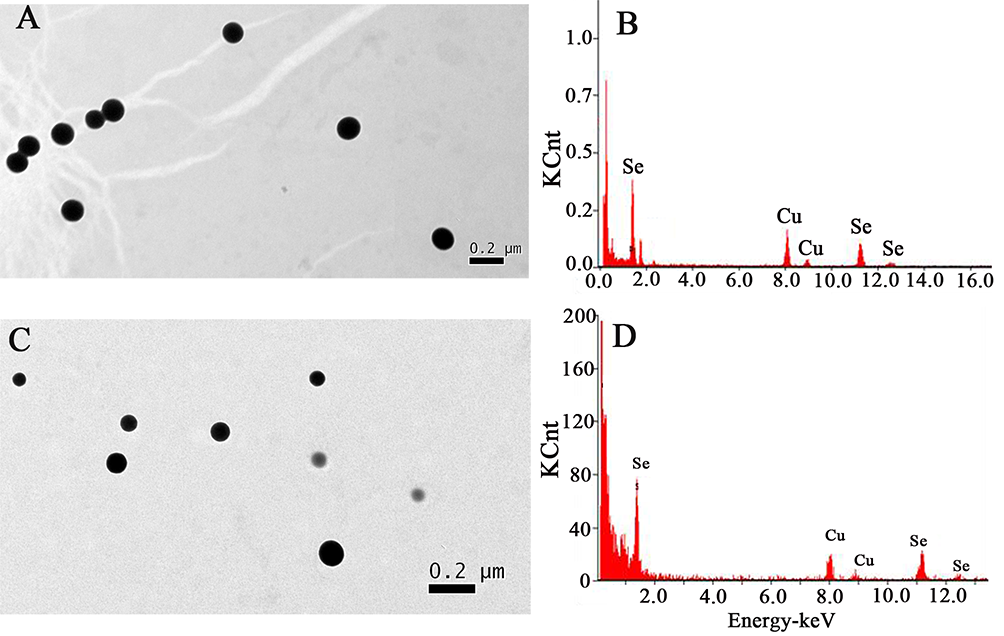

Supplement: Figure S1 — TEM images of (A) CheSeNPs [the ratio of the concentration of sodium selenite (Na2SeO3) to sodium thiosulfate (Na2S2O3) is 1:4] and (C) BioSeNPs (formed by strain HX2 with 15 mM Na2SeO3 for 48 h) and EDX spectra of the chemosynthesized nanoparticles (B) and biosynthesized nanoparticles (D). [file Image_1.TIF]

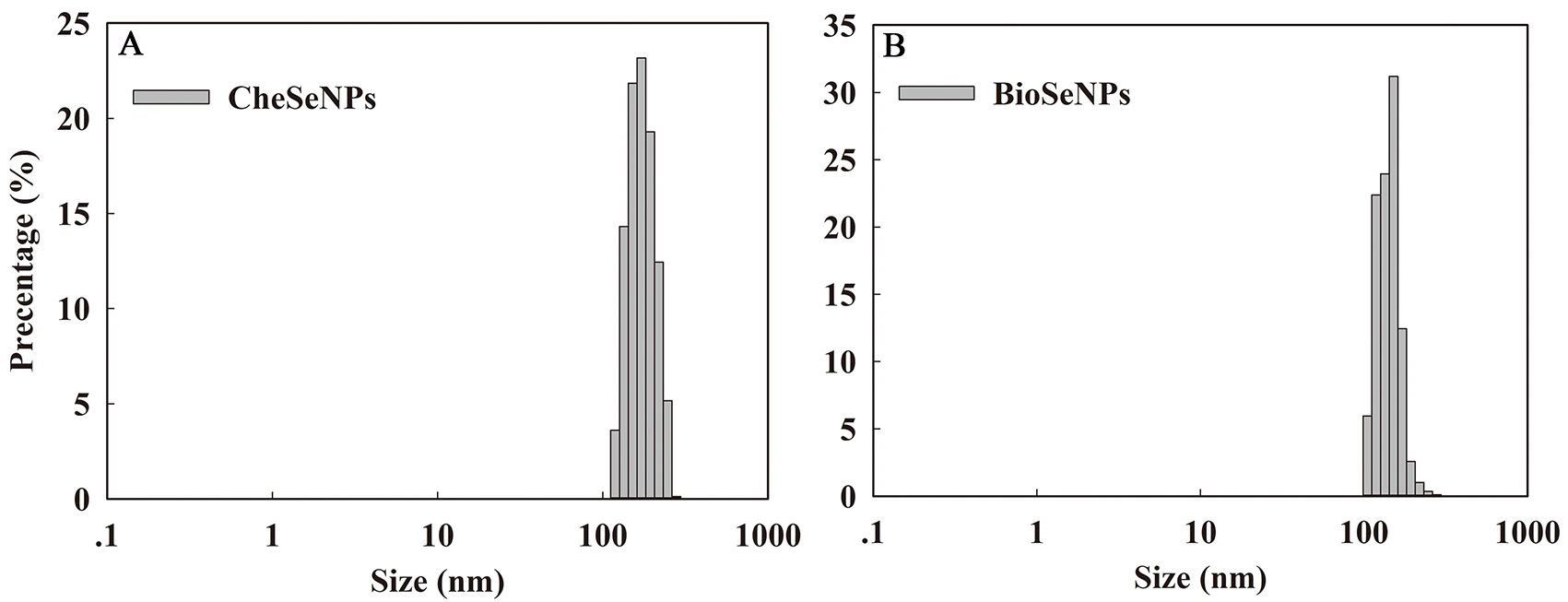

Supplement: Figure S2 — Hydrodynamic diameter of (A) CheSeNPs and (B) BioSeNPs. [file Image_2.TIF]

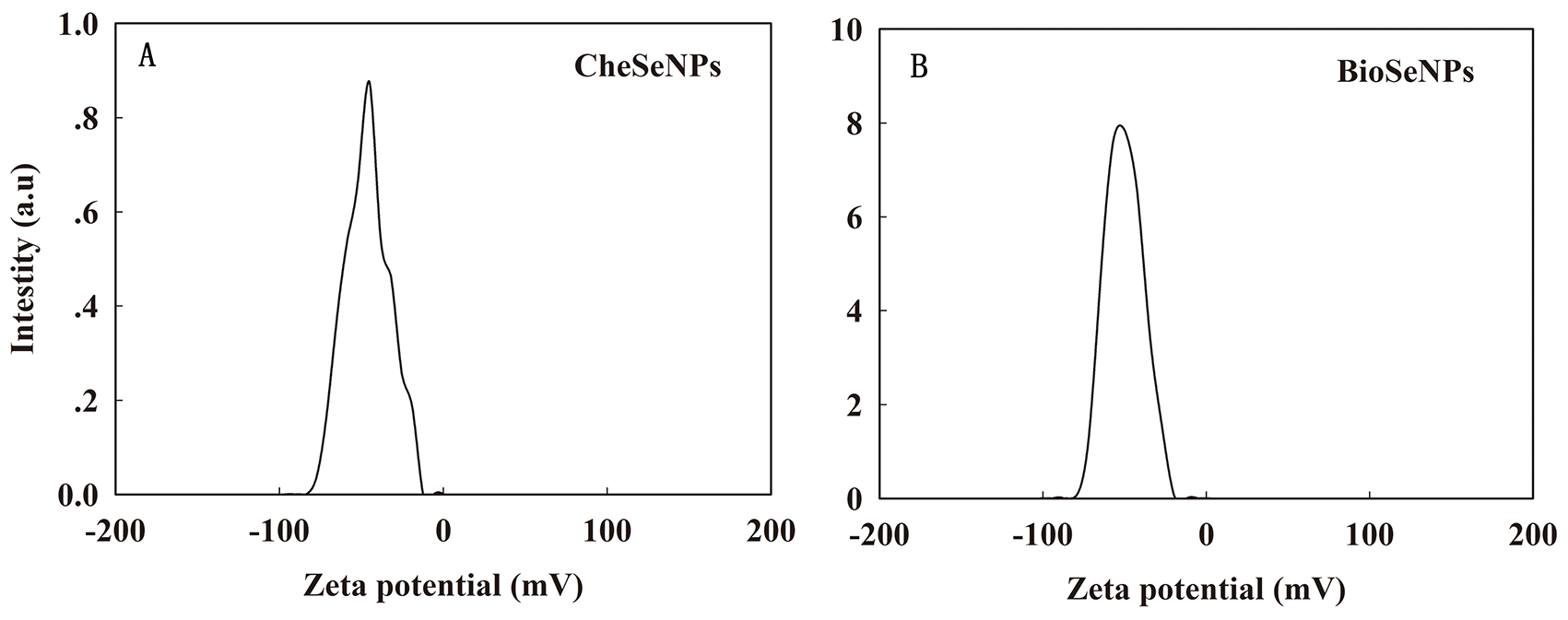

Supplement: Figure S3 — Zeta potential of (A) CheSeNPs and (B) BioSeNPs. [file Image_3.TIF]

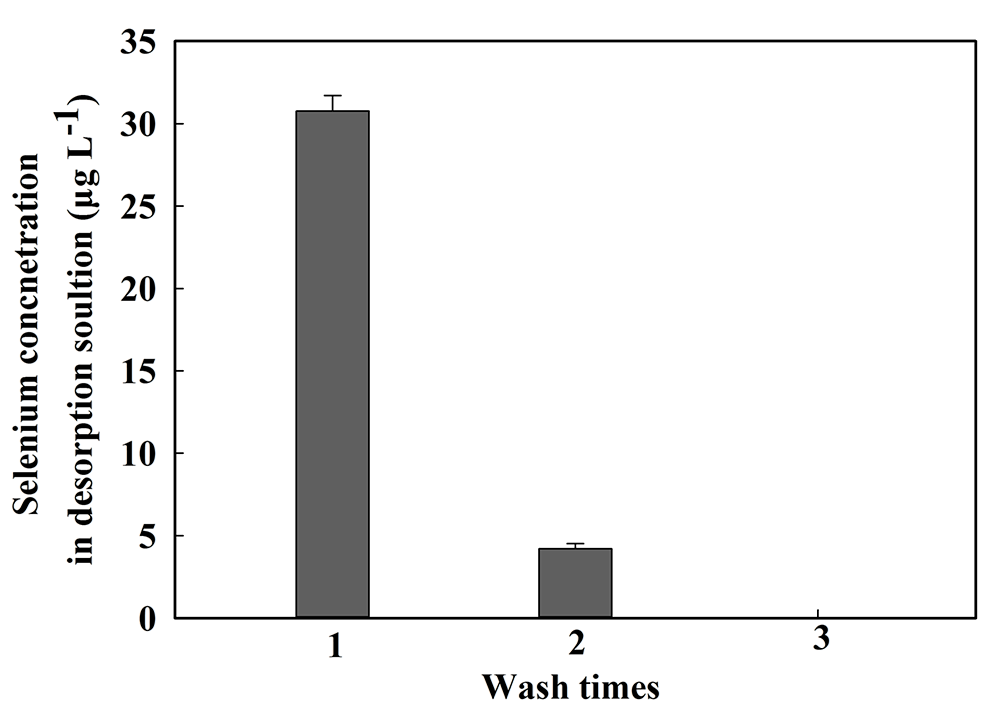

Supplement: Figure S4 — Concentration of Se in the desorption solution with the wash times. [file Image_4.TIF]

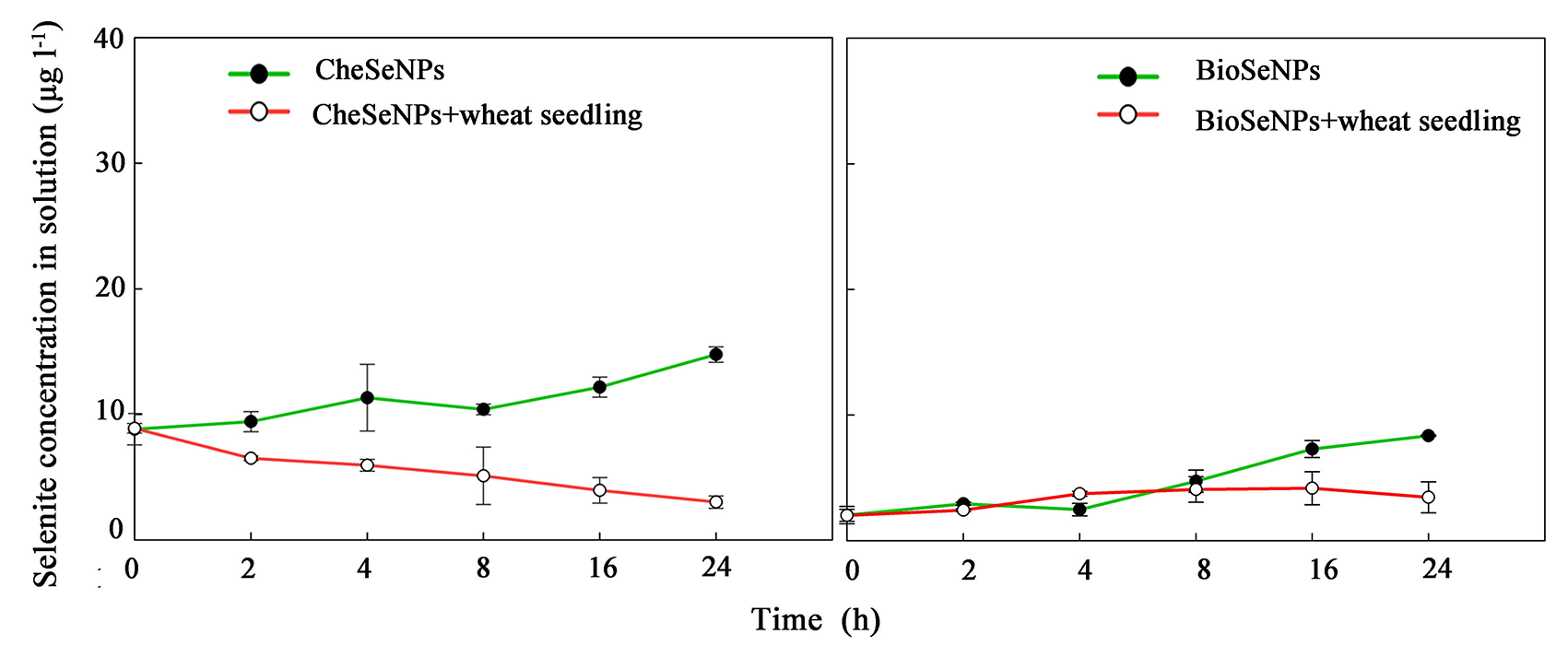

Supplement: Figure S5 — Selenite concentration in SeNPs treatments solutions (CheSeNPs; BioSeNPs). [file Image_5.TIF]
