# Supplementary material for: Absorption and Bio-Transformation of Selenium Nanoparticles by Wheat Seedlings (Triticum aestivum L.)
Source: Front Plant Sci. 2018 May 14;9:597. doi: 10.3389/fpls.2018.00597 (PMC5960721; doi:10.3389/fpls.2018.00597)
Supplement: Table S1 — ICP-MS parameters for analysis of Se species in wheat roots and shoots. [file Table_1.doc]

Table S1 ICP-MS parameters for analysis of Se species in wheat roots and shoots.

| Operational Condition of Agilent 7700x ICP-MS | |
| --- | --- |
| RP power | 1550 W |
| Carrier gas flow rate | 650 mL min^˗1^ |
| Make up flow rate | 450 mL min^˗1^ |
| Nebulizer type | MicroMist |
| Spray chamber type | Quartz impact bead |
| Date acquisition mode | Time resolved analysis |
| Isotopes monitored | ^78^Se |
