# Supplementary material for: Absorption and Bio-Transformation of Selenium Nanoparticles by Wheat Seedlings (Triticum aestivum L.)
Source: Front Plant Sci. 2018 May 14;9:597. doi: 10.3389/fpls.2018.00597 (PMC5960721; doi:10.3389/fpls.2018.00597)
Supplement: Table S2 — Extraction efficiency of selenium in wheat roots and shoots by protease XIV. [file Table_2.doc]

Table S2 Extraction efficiency of selenium in wheat roots and shoots by protease XIV. Mean values (n=3) ± standard error (SE).

| item | | Selenite | | | CheSeNPs | | | BioSeNPs | |
| --- | --- | --- | --- | --- | --- | --- | --- | --- | --- |
|  |  | shoot | root | | shoot | root | shoot | | root |
| total Se content in plant μg g^˗1^ | 11.0±1.3 | | 29.3±4.9 | 13.0±0.3 | | 32.2±1.2 | 3.5±0.2 | | 13.8±1.9 |
| extraction Se content μg g^˗1^ | 5.6±1.2 | | 16.3±2.1 | 6.6±1.6 | | 19.3±1.2 | 1.2±0.3 | | 6.9±1.0 |
| extraction efficiency %* | 51.2±5.1 | | 55.6±2.1 | 50.7±10.9 | | 60.1±4.2 | 33.4±8.2 | | 50.2±0.7 |

*Extraction efficiency was calculated by the extraction Se content / the total Se content in plant×100%.
